# The Association between Serum Resistin Level, Resistin (−420C/G) Single Nucleotide Variant, and Markers of Endothelial Dysfunction, including Salt Taste Preference in Hypertensive Patients

**DOI:** 10.3390/nu14091789

**Published:** 2022-04-24

**Authors:** Katarzyna Musialik, Ewa Miller-Kasprzak, Marta Walczak, Leszek Markuszewski, Paweł Bogdański

**Affiliations:** 1 Department of Treatment of Obesity, Metabolic Disorders, and Clinical Dietetics, Poznan University of Medical Sciences, Szamarzewskiego St. 82/84, 60-569 Poznan, Poland; kasia-musialik@wp.pl (K.M.); pawelbogdanski@ump.edu.pl (P.B.); 2 Department of Internal Medicine, Metabolic Disorders and Hypertension, Poznan University of Medical Sciences, Szamarzewskiego St. 82/84, 60-569 Poznan, Poland; walczak_marta@interia.pl; 3 Department of Medicine, Faculty of Medical Sciences and Health Science, Kazimierz Pulaski University of Technology and Humanities in Radom, Malczewskiego 29, 26-600 Radom, Poland; leszekmarkuszewski@gmail.com

**Keywords:** resistin polymorphism, hypertension, obesity, salt intake, uric acid, arterial stiffness, endothelial dysfunction

## Abstract

**Background:** Resistin action links to conditions such as diabetes, obesity, but its role in hypertension is less well understood. This study aimed to estimate the relationship between resistin (−420G/C) single nucleotide variant (SNV) and markers associated with endothelial dysfunction in hypertension. **Methods:** The study enrolled 162 hypertensive patients (HT) and 165 non-hypertensive (NHT) patients. Resistin serum concentration was estimated with immuoenzymatic assay. Anthropometric measurements, blood pressure and arterial stiffness index (SI), uric acid (UA) serum concentration, and salty taste preference of normal (NS) or high (HS) were assessed in the study. Genotyping was achieved by polymerase chain reaction-restriction fragment length polymorphism. **Results:** Resistin concentration and SI do not differ significantly between HT and NHT individuals; UA significantly increased in HT subjects. Resistin, UA, and SI did not differ among particular resistin genotypes in HT, NHT, NS, or HS groups. GG and CG genotypes were more frequent (OR 1.57 (95% CI; 1.01–2.43); *p* = 0.04) in hypertensive individuals than the NHT group, but less frequent (OR 0.58 (95% CI; 0.37–0.91); *p* = 0.01) in HS patients compared to NS individuals. Concerning HT patients with different salt preferences, GG + CG genotypes were less frequent (OR 0.50 (95% CI; 0.26–0.97); *p* = 0.04) in the HS group than in NS individuals. HT carriers of GG and CG genotype have significantly increased UA concentrations compared to the respective NHT subjects. HS individuals carrying GG and CG genotypes have higher SI values than the NS group. Allele G of SNV (−420G/C) adjusted for age, BMI, serum resistin, UA concentration, salt taste preference, SI, and HR values increased the risk of developing hypertensive phenotype 1.8 fold. **Conclusions:** Resistin SNV (−420G/C) is related to several markers associated with endothelial dysfunction, including salt taste preference in hypertensive patients.

## 1. Introduction

The global prevalence of hypertension in adults is around 30–45% [1]. Among people aged >60 years, HT is more common, with a prevalence of over 60% [2]. Due to populations aging, spreading more sedentary lifestyles, and increasing body weight, HT’s prevalence will continue to rise. It is estimated that the number of people with HT will increase, reaching close to 1.5 billion by 2025 [3]. HT is a significant risk factor for cardiovascular diseases and a contributing factor to shortening life expectancy [4]. The relationship between dietary salt intake, blood pressure (BP) values, and cardiovascular risk is still being discussed. Based on their research analysis, Alderman and Cohen demonstrated that sodium intake below 2.5 g/d and above 6 g/d may be a J-curve—meaning that both too little and too much sodium consumption may be associated with an increase in cardiovascular risk [5]. It is estimated that limiting salt intake to 4.6 g/d reduces BP by 5.0/2.7 mmHg. There is evidence for salt-sensitive and salt-resistance individuals developing hypertension; however, this phenomenon’s exact mechanism remains unclear [6]. Up to 50% of normotensive individuals can be salt-sensitive compared to 75% of hypertensive subjects showing salt sensitivity depending on age, obesity, renal failure, or ethnicity [7]. Both body mass index (BMI) and waist circumference are strongly connected with BP level. The influence of factors occurring in obesity on the development of arterial hypertension is related to the retuning of the sympathetic and endocrine systems. It is estimated that a weight loss of 10 kg may reduce systolic blood pressure (SBP) by about 5–20 mmHg [8]. A high salt diet is associated with endothelial dysfunction [9]. The aldosterone production known to conduct sodium reabsorption in the kidneys may be regulated by factors released from adipose tissue [6]. The issue that adipose tissue is an active source of secreted hormone-like molecules called adipokines changes the view of the whole process of fat tissue metabolism. The number of newly described adipokines has been growing in recent years. One of these molecules is resistin, a cysteine-rich polypeptide encoded by the *RETN* gene located on chromosome 19p13.2. The expression of resistin is not restricted only to adipose tissue; in humans, its rich source is bone marrow and macrophages [10]. As a risk factor for hypertension, serum resistin was previously postulated by Zhang et al. in a metanalysis of 14 case–control studies that included individuals with hypertension [11]. The mechanism underlying the association between resistin and hypertension remains unclear. Hypertension is also related to the dysfunction of the endothelium layer. Other markers are associated with hypertension, such as one of the end products of purine metabolism, uric acid (UA). Hyperuricemia has emerged as one of the most substantial risk factors for the development of pre-hypertension, primary hypertension, and resistant hypertension [12,13,14,15]. Some experimental and clinical studies showed that lowering serum UA level improves SBP and diastolic blood pressure (DBP). Similarly, arterial stiffness and the rebound phenomenon are the most important factors in increasing SBP and pulse pressure (PP) in aging societies. Arterial stiffness and central PP should be investigated in patients with hypertension because these phenomena are of prognostic value in cardiovascular events [16]. The stiffness index (SI) is defined as the quotient of the patient’s height and the propagation time of the reflected wave [17].

Resistin is a molecule believed to play a proinflammatory role in hypertension and coronary artery diseases (CAD) [18]. Resistin (−420G/C) single nucleotide variant (SNV) has been studied in several pathological conditions, including colorectal cancer, ischemic stroke, metabolic syndrome, insulin resistance, and non-alcoholic fatty liver disease [19,20,21,22,23]. Due to promoter localization, the resistin SNV (−420G/C) was associated with serum resistin level; however, some authors failed to demonstrate such a relationship [18,20,24,25]. The impact of allele G of resistin SNV (−420G/C) as a risk factor in several conditions has been frequently considered. However, the data about the involvement of this particular SNV in hypertension are limited.

Our study aim was to estimate the putative association between serum resistin concentration, resistin SNV (−420G/C) distribution, and markers related to endothelial dysfunction, including salt taste preference in hypertensive patients.

## 2. Materials and Methods

### 2.1. Study Population

The local Bioethics commission approved the study (No. 359/15, No. 1312/18). The study was performed in agreement with the Helsinki Declaration. Patients enrolled in the study in the Metabolic Disorders Outpatient Clinic at Clinical Hospital of Lord’s Transfiguration in Poznan, Poland. After accepting the study’s primary purpose, the complete consent form was collected from each patient. A total of 162 hypertensive individuals (HT) and 165 non-hypertensive patients (NHT) were enrolled in the study. The inclusion criteria involved: ≥18 years old, stable body mass (±3 kg) over the previous month, newly diagnosed hypertension or lack of hypertension for the HT and NHT group, respectively. The exclusion criteria were as follows: type-2 diabetes mellitus, kidney diseases, clinically significant impaired liver function, a chronic or acute state of inflammation, tumor disease, alcohol or nicotine abuse.

### 2.2. Anthropometric and Physiological Parameters

All study participants were advised to maintain a normal diet, avoid consuming alcohol, caffeine, and not perform intensive physical exercises 24 h before the examination. Each patient was asked to respond to the personal preference for salty taste in their routine dietary habits expressed on a dichotomous scale (0—normal salty taste preference (NS) or 1—high salty taste preference (HS; like adding additional salt to dishes). The weight and height of each patient who qualified for the study were assessed using a RADWAG WPT 100/200 OW stadiometer with an electric scale of the accuracy of 0.1 kg and 0.5 cm, respectively. After an overnight fast, patients were examined in the morning, while wearing light clothes. Waist circumference was assessed midway between the costal arch and the upper iliac crest using standard medical instruments with an accuracy of 0.5 cm. BMI was calculated based on BMI’s standard equation to body weight (kg)/height (m²). The measurements of blood pressure in the studied population were performed according to the recommendations of the European Society of Cardiology (ESC) and the European Society of Hypertension (ESH). Patients were asked to rest for five minutes before measurement in a sitting position. The cuff was selected strictly according to the arm circumference (13 cm long or wider) of a particular patient and situated at the heart’s height during the measurement procedure on the right arm. The measurements were taken three times, separated by a two-minute interval, using an ESH-validated electronic sphygmomanometer (705IT, Omron Corporation, Kyoto, Japan), to calculate mean SBP, DBP and heart rate (HR) value. Alongside pulse waveform parameters, the stiffness index (SI), reflection index (RI), and peak to peak time (PPT), were obtained and calculated by the use of the pulse SphygmoCor Px (Atcor Medical Blood Pressure Analysis System, Sydney, Australia) from the individual’s finger according to the manufacturer’s protocol.

### 2.3. Biochemical and Genotyping Analysis

Blood samples were collected from each participant in the morning during fasting. Resistin concentration was estimated from serum collected samples using an immunoenzymatic method using a commercially available kit (FineTest, Wuhan, China). Uric acid serum concentrations (UA) were obtained from a commercial laboratory through standard enzymatic methods. Samples collected in ethylenediaminetetraacetic acid-containing peripheral blood were used to isolate DNA by Master Pure DNA Purification Kit (Epicentre, Lucigen, Middleton, WI, USA). Genomic DNA was amplified with specific forward *5′TGTCATTCTCACCCAGAGACA3′* reverse *5′TGGGCTCAGCTAACCAAATC3′* primers complementary to the sequences accompanied the site of SNV (−420G/C) polymorphism (*rs1862513*) as described previously [26]. Genotyping was performed by restriction length polymorphism of polymerase chain reaction method (PCR-RFLP). The PCR product size was 534 base pair (bp) in length. Fragments obtained by *BBsI* restriction endonuclease (Thermo Fisher Scientific, Waltham, MA, USA) cleavage of C allele PCR product correspond to 327 bp and 207 bp bands. In contrast, the G allele-specific PCR product has no recognition site for *the BBsI* enzyme (Acc. no. NG_023447). Fragments were visualized on 3% agarose gel with Midori Green (Nippon Genetics, Tokyo, Japan). About 10% of randomly selected samples were re-genotyped to confirm the reproducibility of the assay.

### 2.4. Statistical Analysis

Statistical assessments were conducted using Statistica 13.0 software (Statsoft, Tulsa, OK, USA). Values were present as a mean with standard deviation (SD). The normal distribution of variables was checked by the Shapiro–Wilk test. The distribution of genotypes was evaluated by a contingency table analysis chi-squared (χ^2^) test. The odds ratio (OR) and 95% confidence intervals (95% CI) were calculated. Agreement with the Hardy–Weinberg equilibrium was estimated using the snphwe package in Python 3.10. According to the compliance or non-compliance of variables with the Gaussian curve, the Student’s *t*-test and analysis of variance (ANOVA) for unrelated variables or Mann–Whitney test and Kruskal–Wallis test for unrelated variables with the post hoc test were applied, respectively. The Pearson correlation test was used for normally distributed values, and Spearman’s rank correlation test was used for variables lacking the normal distribution to estimate the relationship between examined parameters. A multivariable logistic regression model was applied to determine the influence of resistin SNV (-420 C/G) on hypertension phenotype. The power of the study was calculated using Genetic Association Study (GAS) Power Calculator. The level of *p* < 0.05 was considered statistically significant. 

## 3. Results

According to the design of the study, both HT and NHT groups differ in SBP (*p* < 0.001), DBP (*p* < 0.001), and HR (*p* < 0.01) values. A significant difference between groups was noticed in body mass (BM; *p* < 0.001), BMI (*p* < 0.01), WC (*p* < 0.01), although a comparable percentage of HT and NHT patients (about 66%) were obese. Among arterial stiffness parameters, only PPT in HT patients presented significantly decreased values compared to the NHT controls (*p* = 0.01). SI and RI parameters did not differ between groups. Resistin serum concentration did not differ significantly between HT and NHT groups. UA serum concentration increased in HT compared to NHT controls (*p* = 0.02). The reported salty taste preference percentage was comparable between the studied groups (about 47%). Both HT and NHT groups were similar in age and sex distribution. The characteristics of the studied population are given in Table 1. 

The observed genotype frequencies for SNV (−420G/C) were consistent with Hardy–Weinberg equilibrium (*p* = 0.62). Frequencies for C and G alleles in the entire population were 0.66 and 0.34, respectively, consistent with a mean allele frequency (MAF) TopMed population reported in the Ensembl database for the rs1862513 resistin variant. The power of the case–control study was estimated to be 0.87 in the dominant model of inheritance with alpha = 0.05.

Further analysis was performed by splitting participants into HT and NHT groups according to hypertension phenotype and HS and NS groups according to high or normal salt taste preference. Patients from the HT and NHT groups did not differ significantly in the frequency of particular genotypes according to the additive inheritance model (Table 2). 

We divided the studied patients into the GG + GC and CC genotype-possessing individuals according to the dominant inheritance model to perform further comparisons. We found that regarding GG + GC vs. CC individuals, the distribution differed between HT and NHT groups, and GG and CG genotypes were more frequently present in HT individuals (OR = 1.57 (95% CI; 1.01–2.43); *p* = 0.04) (Table 2). Conversely, GG and GC genotypes appear significantly less frequently (OR = 0.58 (95% CI; 0.37–0.91); *p* = 0.01) in salty-taste-preferring (HS) individuals compared to NS subjects (Table 3). 

The frequencies of genotypes were similar among HT and NHT women and respective men groups (Table S1). Similarly, no statistically significant differences were noticed in the distribution of particular genotypes and GG + GC vs. CC genotypes in the salty--taste preferring HS group according to the hypertension phenotype (Table 4). 

HT subjects from NS group alone possess GG and GC genotypes more frequently (OR 1.86 (95% CI; 0.99–3.51); *p* = 0.04; Table 5). 

When considering the HT group alone, GG + GC individuals presented high salty taste preference less frequently (OR = 0.50 (95% CI; 0.26–0.97); *p* = 0.04) than CC genotype harboring subjects (Table 6), while no such result was observed in the NHT group among HS and NS individuals (Table 7). 

The serum concentration of resistin was similar in the studied population when evaluated according to hypertension phenotype or salty taste preference, which was compared among genotypes and GG + GC vs. CC individuals. Similarly, endothelium-related parameters such as serum UA concentration and SI values did not differ significantly among particular genotypes or groups (Table 8).

Further analysis was performed separately in groups bearing GG + GC genotypes and CC genotypes. Hypertensive individuals from the GG + GC group did not differ in the serum resistin concentration compared to NHT subjects. Similarly, no differences were found when CC genotype-possessing subjects with HT and without HT were compared (Table 9). 

Conversely, UA concentration significantly increased in HT individuals possessing the GC or GG genotype (*p* = 0.03) compared to NHT counterparts. Only CC genotype-bearing patients with HT revealed significantly increased SI value compared to NHT subjects with the same genotype pattern (*p* = 0.03). Concerning salty taste preferences, HS individuals who carried GC or GG genotype showed significantly higher SI values than NS subjects with identical genotypes (*p* = 0.04; Table 9). 

Multiple correlations were found between the studied parameters. In the whole population resistin concentration positively corelated with SI value (r = 0.17). UA corelated positively with Age (r = 0.17), BM (r = 0.35), WC (r = 0.36), SI (r = 0.20), SBP (r = 0.17) and DBP (r = 0.11) and SI corelated with Age (r = 0.35), WC (r = 0.15), resistin (r = 0.17), SBP (r = 0.24), DBP (r = 0.17) and UA (r = 0.20). 

In all participants possessing the CC genotype, we found correlations between UA and BM (r = 0.40), and UA and DBP (r = 0.18), and SI and Age (r = 0.28).

In HT individuals carrying the CC genotype, the correlations between resistin and age (r = 0.29) and UA and BM (r = 0.30), DBP (r = 0.28), WC (r = 0.33) as well as and between SI and Age (r = 0.35) were noticed. 

In NHT subjects carrying the CC genotype, a correlation between resistin and SI (r = 0.29), the correlation between UA and BM (r = 0.50) and WC (r = 0.54) as well as SI and resistin (r = 0.29) and SI and Age (r = 0.25) were demonstrated. 

There was no relationship between resistin and studied parameters in GG + CG and CC genotype-carrying individuals from HS and NS groups.

In HS subjects with the CC genotype, we noticed positive correlations between UA and BM (r = 0.54), DBP (r = 0.28), and SI with Age (r = 0.29), SBP (r = 0.32), DBP (r = 0.21). 

In NS individuals carrying the CC genotype, we found a correlation between UA and BM (r = 0.24) and SI with Age (r = 0.26).

In all individuals harboring GG or CG genotype, resistin positively correlated with DBP (r = 0.17) while UA corelated with Age (r = 0.22), BM (r = 0.29), SI (r = 0.24) and SBP (r = 0.23). In GG or GC genotype carrying group we also found a positive correlation between SI and Age (r = 0.40), SBP (r = 0.30) and UA (0.24). 

Hypertensive subjects with GG or GC genotype showed a correlation between UA and SI (r = 0.24) and SI and Age (r = 0.27), while in NHT individuals possessing GG or CG genotype a correlation between resistin and DBP (r = 0.29), and between UA and BM (r = 0.40), SBP (r = 0.28) and age (r = 0.27) along with SI with Age (r = 0.54) and SBP (r = 0.35) were observed. 

Concerning GG + GC carrying HS subjects, several correlations were noticed, particularly between UA and BM (r = 0.29) and SI (r = 0.40) and SI with Age (r = 0.65), SBP (r = 0.40) and UA (r = 0.40). In NS GG + GC group the correlations between UA, correlation with BM (r = 0.23), SBP (r = 0.24) and SI with SBP (r = 0.23) and DBP (r = 0.24) were confirmed (Table 10). 

The multivariable logistic regression model adjusted for several confounders, among which were age, BMI, serum resistin and UA concentration, salt taste preference, and SI, and HR values showed that the G allele of SNV (−420G/C) significantly increases the risk of hypertension (OR 1.89 (95% CI; 1.01–3.52); *p* = 0.04). Both endothelial-function-related parameters, e.g., SI (OR 1.22, (95% CI; 1.06–1.4); *p* = 0.005) and HR (OR 1.03 (95% CI; 1.01–1.06); *p* = 0.006) remained independent predictors of hypertension status in above model.

## 4. Discussion

Hypertension is an emerging civilization disease. The impact of the inflammation process on hypertension has been subject to debate [18]. Hypertension is accompanied by several metabolic-related disorders, including insulin resistance, type 2 diabetes mellitus, and obesity. Dietary habits, including salt overuse, are also associated with the development of hypertension [5,6]. Hypertension has been linked to several pathological conditions related to endothelial dysfunction, including adiposity, changes in UA metabolism, and arterial stiffness [13,17]. The exact role of resistin in hypertensive patients has not been elucidated. 

Our study, for the first time, to the best of our best knowledge, demonstrated that resistin SNV (−420G/C) is related to hypertension and may interplay with markers of endothelial dysfunction, including serum UA concentration, SI value, and salt taste preference in hypertensive patients. Resistin in humans is mainly produced by macrophages and could affect several tissues as a secreted cytokine [18]. Recently, studies have shown the association between circulating resistin levels and hypertension phenotype [27,28]. The results of initial efforts were conflicting, with some but not all studies identifying a significant correlation between serum resistin level and hypertension incidence. Niaz et al. demonstrated a progressive increase in serum resistin levels in hypertensive and coronary artery disease patients compared to normal subjects [18]. Hsu et al. showed that a high serum resistin level could be an independent predictor of peripheral artery disease in patients with hypertension [29]. The serum resistin concentration did not differ between HT and NHT individuals in our study. Similar to our results, Bielecka-Dabrowa et al. [30] found no significant difference in serum resistin levels between patients with newly diagnosed hypertension and non-hypertensive controls. It has also been suggested that a high salt diet might be involved in the course of hypertension. Salt-sensitive hypertension is proposed to be associated with oxidative stress and inflammation processes and is though to disturb negatively charged glycocalyx of vascular endothelial cells by NaCl exceeding 5% [7]. Salt sensitivity hypertension has been observed in 40–75% of hypertensive individuals compared to 25–50% of normotensive subjects. Among hypertensive individuals, there are salt-sensitive persons with an increase in blood pressure of more than 10% after salt consumption and salt-resistant ones with less than 10% increase in blood pressure after salt intake [7]. It has been demonstrated that resistin expressed by monocytes is reduced in patients treated with loop diuretics, and loop diuretics may regulate resistin synthesis [31]. In the animal study, serum resistin concentration did not significantly differ between C57BL6 mice fed a high-fat or low-fat plus high-salt diet [32]. In a cross-sectional study performed by Cha et al., a high-salt diet intake was accompanied by higher plasma resistin levels in overweight or obese adults with poor sleeping behavior [33]. Conversely, the intervention study with dietary sodium manipulation conducted on hypertensive patients by Vaidya et al. [34] showed that a low sodium diet was associated with a higher plasma resistin level compared to a high sodium loaded diet. It suggested that dietary sodium decreases plasma resistin levels via the action of the renin-angiotensin-aldosterone system (RAAS). Notably, in our study, almost 46% of hypertensive subjects tended to overuse salt in dishes, which may result in the modulation of primary serum resistin levels and the subsequent absence of significant difference in the resistin concentration between HT and NHT individuals. 

The discrepancy data among studies estimating the association of resistin concentration with the hypertension phenotype may derive from not taking into account particular genetic variants of resistin directly related to resistin expression level. Resistin SNV (−420G/C) is located in the promoter region of the *RETN* gene, and plasma resistin is reported to be highest in GG genotype carrying individuals with diabetes compared to remaining SNV (−420G/C) genotypes [22]. In the study performed on Korean subjects with and without type 2 diabetes, Cho et al. also showed that plasma resistin concentrations are different in particular resistin SNV (−420G/C) genotypes. The CC genotype carriers presented decreased resistin concentration compared to CG- and GG-harboring individuals [24]. Resistin SNV (−420G/C) was also accompanied by increased resistin serum levels in Malaysian men in the same pattern mentioned above [35]. Tsukahara et al. also proved that serum resistin elevation proceeds according to a genotype-dependent manner in Japanese diabetic patients [20]. However, there were no differences in serum resistin concentration between HT vs. NHT or HS vs. normal salt preferring (NS) subjects among particular resistin SNV (−420G/C) genotypes in our study. In line with our study, Norata et al. did not observe any significant differences in plasma resistin levels among individuals possessing particular resistin SNV (−420G/C) genotypes and participating in the cohort study [25]. Interestingly, in our study, individuals with GG or CG genotypes were almost twice as frequently at risk of being hypertensive as those with CC genotype. The study by Kumar et al. performed on adult women with metabolic syndrome found that CG and GG genotypes possessing women presented significantly higher values of SBP and DPB as compared to women harboring the CC genotype [23]. In the study by Miyamoto et al. [36], allele G of resistin SNV (−420G/C) significantly correlated with high DBP in Japanese men from an urban cohort. Tsukahara et al. performed a study on the Japanese population and documented that individuals harboring CG and GG genotypes were at a greater risk of stroke than CC individuals [20]. On the other hand, Boumaiza et al. did not present any differences when comparing blood pressure among individuals with particular genotypes [37]. Authors suggested that GG and GC genotypes of the resistin variant are associated with the risk of metabolic syndrome in the Tunisian cohort [37]. However, there was no difference in SBP and DBP values among particular resistin SNV (−420G/C) genotypes in obese participants [37]. Recently, resistin has been found to influence the expression of neuropeptide Y, a potent orexigenic factor in the central nervous system [38]. In some studies, the G allele was associated with an obese phenotype, while other authors did not find any relationship between obesity and the G allele [39]. Beckers et al. did not observe an association between their studied resistin genetic variants, among them SNV (−420G/C), and obesity in the female population [40]. Mattevi et al. suggested that the SNV (−420G/C) variant had gender-specific effects on BMI, and females possessing the G allele had lower BMI values than CC genotype carrying women [41]. Although about 66% of individuals from each HT and NHT group were classified as obese in our study, the logistic regression model adjusted for BMI demonstrates the significant association of the G allele with hypertension. 

In our study, individuals with GC and GG the resistin genotype of SNV (−420G/C) preferred salting dishes less frequently than CC genotype harboring individuals. The ability to influence SNV (−420G/C) resistin variant on diet response has been recently documented in the study by Aberle et al. [38]. Authors found that carriers of the G allele responded better to a low-calorie diet and improved the metabolic tract as compared to CC-harboring individuals. After three months of maintaining a low-fat diet, carriers of at least one G allele of the SNV (−420G/C) resistin variant lost more weight and presented decreased triglycerides as compared to the wild-type genotype group [38]. Recently, Noumi et al. performed an interesting study to estimate serum resistin concentrations concerning the preferred diet in the Japanese population [42]. The authors genotyped SNV (−420G/C) polymorphism and found that serum resistin and n-3 polyunsaturated fatty acids intake was inversely associated and that correlation was strongest in GG genotype individuals [42]. The abovementioned studies suggest the possible influence of resistin and its genetic variant SNV (−420G/C) on the aspect of nutrition. High dietary salt intake has been linked to the inflammation process [6,7,43]. Tang et al. described that GG- and GC-genotype-carrying individuals with CAD presented an elevated level of high sensitive C-reactive protein compared to CC patients [44]. GG genotype and G allele of SNV (−420G/C) may be linked to autoinflammatory-related Kawasaki disease [45]. It has also been found that patients with GG and GC genotypes have an increased risk of CAD compared to CC carriers [46]. Bahia et al. found that resistin correlates with endothelium-dependent vasodilation in individuals with metabolic syndrome and suggested that resistin may influence endothelial dysfunction [47]. Recently, Wang et al. demonstrated that serum resistin concentration is an independent predictor of aortic stiffness estimated as carotid-femoral pulse wave velocity in patients with CAD. Patients characterized by higher aortic stiffness presented elevated serum resistin levels compared to the lower aortic stiffness group [48]. Similarly, the CC-genotype-possessing individuals from the non-hypertensive group presented a significant positive correlation between the SI value and resistin in our study. A salt diet leads to a change in the negative charge of endothelial cells’ glycocalyx layer [9]. Chronic exposure to 5% NaCl leads to hyperosmotic stress, endothelial cell shrinkage, and increased vascular layer permeability [7]. In our study, subjects with CG and GG genotypes who prefer salting dishes (HS) presented significantly higher SI values than the NS group. It might suggest that the G allele may predispose patients to salt diet-induced endothelial dysfunction. We also demonstrated a positive SI and UA correlation that concerns only the CG + GG genotype group that was significant in HT individuals and subjects who prefer salt taste (HS). Recently, the link between hyperuricemia and arterial stiffness has been discussed by Albu et al. [49]. UA is related to RAAS action and endothelial dysfunction and is proposed to be involved in endothelium homeostasis. Wang et al., in an intervention study with a salt diet, demonstrated that UA plasma levels decreased after the high salt intake in Chinese adults [50]. The authors explained that sodium nephron reabsorption related to RAAS action is accompanied by urate reabsorption in the other part of the nephron. They suggest that a high salt diet modified UA plasma levels in salt-sensitive individuals, indicating UA’s role in the pathogenesis of hypertension [50]. Our study shows that UE vs. SI correlation is visible in allele G-possessing individuals. Interestingly, we also found that in GG + CG genotype patients, the concentration of UA is significantly higher in HT than in NHT individuals. No such difference was present in the CC-genotype-harboring individuals. Regarding the HS group, SI was found to correlate with UA levels only in individuals possessing CG and GG genotypes which suggest salty taste preference and resistin SNV (−420G/C) influence the modulation endothelium dysfunction markers in the studied population. This finding may suggest that environmental factors, including salty diet preference, may cumulate the risk of endothelial dysfunction in patients possessing GG or CG genotype of resistin SNV (−420G/C). CC genotype harboring individuals of resistin SNV (−420G/C) revealed correlations of UA with BM and SBP. The UA concentration has been described as a strong predictive marker of the development of obesity [14]. In our study, the SI value was also found to be significantly increased in HT CC-genotype-possessing individuals compared to non-hypertensive CC genotype bearing subjects. However, in our multivariable logistic regression model SI value was an independent risk factor for hypertension and is not related to the presence of the resistin G allele that might explain obtained results. HT subjects carrying the CC genotype also presented a positive correlation between resistin concentration and age. Resistin was found to correlate with age in other studies in African women after abdominal surgery [51] or patients with CAD [48], but no genotyping analysis was there performed. 

Our study has some limitations, mainly relating to the relatively small number of analyzed individuals and the observational character of the study. However, in this study, we mainly focused on the interaction of resistin and its genetic variant with clinical parameters of endothelial dysfunction in the studied population. Further studies may benefit from larger populations, and a haplotype analysis that includes other functional resistin genetic variants and their possible interaction with a salty diet.

Contrary to others, Ukkola et al. showed that in hypertensive patients the CC genotype predisposes individuals to a worse metabolic profile, not GC and GG genotypes of resistin SNV (−420G/C). The authors explained the results using the interactions of this polymorphism with other gene variants and metabolic or environmental factors [52]. Indeed, the impact of resistin SNV (−420G/C) on serum resistin concentration has been recently extended by Onuma et al. to the epigenetic component [53]. Previously, Osawa et al. found that diabetes mellitus susceptibility is linked to GG genotype by SP1/3 transcription factor binding to the *RETN* promoter and increasing its activity [22]. Onuma et al. presented that the *RETN* promoter’s methylation level in peripheral blood cells from Japanese subjects was the highest in CC-genotype-possessing individuals and lowest in GG carriers. The methylation level in genotypes inversely correlates with serum resistin concentration. In vitro assay showed that THP-1 human monocytes that carry CC genotype may undergo a reversed methylation status via the demethylation agent. It causes an upregulation of the *RETN* transcript [53]. Interestingly, NaCl may act as a demethylating agent. In vitro, NaCl induced the DNA hypomethylation of CD4^+^T cells from systemic lupus erythematosus patients [54]. The nutrigenetic component may also be somehow involved in circular resistin level regulation. Preferences for salty taste have been studied regarding genetic variants of the transient receptor potential cation channel subfamily V member 1 [55]. Less is known about the putative involvement of genes not related to taste receptors and sequence variants in particular food including “like” or “dislike”. The fat mass obesity gene *FTO* common sequence variant was recently related to the prevalence of a high fat and high carbohydrate diet [56]. It has also been demonstrated that fibroblast growth factor 21 SNVs are involved in the Emirati population’s salt and sweet taste preferences [56,57]. To the best of our knowledge, the involvement of resistin SNV (−420G/C) in salty taste preference has not been studied. The relationship between resistin, its polymorphism, and markers of endothelial dysfunction in the course of hypertension requires further studies. To draw further conclusions, future studies should consider environmental factors, including patients’ dietary choices and taste preferences.

## Figures and Tables

**Table 1:** Clinical characteristics of the study population.

Parameter	*N*	Hypertensive (HT) *n* = 162	Non-Hypertensive (NHT) *n* = 165	*p*-Value
Female %		70.3%	65.43%	NS ^^^
Age (years)	162/165	60.46 ± 10.81	58.32 ± 12.36	NS *
Body mass (kg)	162/165	91.89 ± 15.61	84.75 ± 14.10	<0.001 *
BMI (kg/m²)	162/165	33.22 ± 4.58	31.38 ± 3.80	<0.01 *
Waist circumference	162/165	107.54 ± 13.58	101.75 ± 13.09	<0.01 *
SBP (mmHg)	162/165	156.16 ± 14.39	127.04 ± 11.32	<0.001 ^#^
DBP (mmHg)	160/162	91.20 ± 11.51	77.54 ± 9.09	<0.001 ^#^
HR	162/162	78.91 ± 14.39	75.50 ± 11.77	0.01 ^#^
SI	143/134	8.83 ± 4.03	8.73 ± 10.03	NS ^#^
PPT (ms)	143/121	217.39 ± 68.08	248.32 ± 63.20	0.01 ^#^
RI (%)	143/119	53.88 ± 22.59	51.79 ± 15.87	NS ^#^
Resistin (ng/mL)	125/134	6.7 ± 3.61	6.52 ± 2.97	NS *
Uric acid (mg/dL)	160/163	5.88 ± 1.32	5.55 ± 1.35	0.02 *
Obesity %		66.03	66.46	NS ^^^
High salty taste preference %		45.45	49.69	NS ^^^

SD: standard deviation; *p*: statistical significance; NS: not significant; *N*: (number of patients), BMI: body mass index; SBP: systolic blood pressure; DBP: diastolic blood pressure; HR: heart rate; PPT: peak-to-peak time; RI: reflection index; SI: stiffness index; * Mann–Whitney U test; ^#^ T-student test. ^ χ^2^ test. All other values are expressed as mean ± SD or percentage.

**Table 2:** Genotype frequencies of SNV (−420G/C) among hypertensive (HT) and non-hypertensive (NHT) individuals.

Total				
Genotype	Hypertensive (HT)			Non-Hypertensive (NHT)
CC	62 (38.51%)			80 (49.08%)
CG	78 (48.75%)			64 (39.26%)
GG	21 (13.04%)			19 (11.6%)
Comparison	χ^2^	*p*-value	OR	(95% CI)
CC/CG/GG	3.74	0.15	-	-
GG + CG/CC	4.04	0.04	1.57	(1.01–2.43)

*p*—statistical significance; OR—odds ratio; χ^2^ test.

**Table 3:** Genotype frequencies of SNV (−420G/C) among high salty taste preference (HS) and normal salty taste preference (NS) subjects.

Total				
Genotype	High Salty Taste Preference (HS)			Normal Salty Taste Preference (NS)
CC	74 (50.00%)			66 (40.24%)
CG	57 (38.51%)			76 (46.34%)
GG	17 (11.49%)			22 (13.41%)
Comparison	χ^2^	*p*-value	OR	(95% CI)
CC/CG/GG	2.99	0.22	-	-
GG + CG/CC	5.49	0.01	0.58	(0.37–0.91)

*p*—statistical significance; OR—odds ratio; χ^2^ test.

**Table 4:** Genotype frequencies of SNV (−420G/C) in high salty taste preference group (HS) according to hypertension phenotype.

High Salty Taste Preference (HS)				
Genotype	Hypertensive (HT)			Non-Hypertensive (NHT)
CC	32 (45.71%)			42 (53.85%)
CG	30 (42.86%)			27 (34.62%)
GG	8 (11.43%)			9 (11.54%)
Comparison	χ^2^	*p*-value	OR	(95% CI)
CC/CG/GG	1.13	0.56	-	-
GG + CG/CC	1.05	0.30	1.4	(0.7–2.49)

*p*—statistical significance; OR—odds ratio; χ^2^ test.

**Table 5:** Genotype frequencies of SNV (−420G/C) in normal salty taste preference group (NS) according to hypertension phenotype.

Normal Salty Taste Preference (NS)				
Genotype	Hypertensive (HT)			Non-Hypertensive (NHT)
CC	28 (33.73%)			38 (46.91%)
CG	42 (50.60%)			34 (41.98%)
GG	13 (15.66%)			9 (11.11%)
Comparison	χ^2^	*p*-value	OR	(95% CI)
CC/CG/GG	3.06	0.21	-	-
GG + CG/CC	3.71	0.04	1.86	(0.99–3.51)

*p*—statistical significance; OR—odds ratio; χ^2^ test.

**Table 6:** Genotype frequencies of SNV (−420G/C) in hypertensive (HT) individuals according to salty taste preference.

Hypertensive (HT)				
Genotype	High Salty Taste Preference (HS)			Normal Salty Taste Preference (NS)
CC	32 (45.71%)			28 (33.73%)
CG	30 (42.86%)			42 (50.60%)
GG	8 (11.43%)			13 (15.66%)
Comparison	χ^2^	*p*-value	OR	(95% CI)
CC/CG/GG	2.36	0.30	-	-
GG + CG/CC	4.14	0.04	0.50	(0.26–0.97)

*p*—statistical significance; OR—odds ratio; χ^2^ test.

**Table 7:** Genotype frequencies of SNV (−420G/C) in non-hypertensive (NHT) individuals according to salty taste preference.

Non-Hypertensive (NHT)				
Genotype	High Salty Taste Preference (HS)			Normal Salty Taste Preference (NS)
CC	42 (53.85%)			38 (46.91%)
CG	27 (34.62%)			34 (41.98%)
GG	9 (11.54%)			9 (11.11%)
Comparison	χ^2^	*p*-value	OR	(95% CI)
CC/CG/GG	0.94	0.62	-	-
GG + CG/CC	1.48	0.22	0.67	(0.36–1.22)

*p*—statistical significance; OR—odds ratio; χ^2^ test.

**Table 8:** Serum concentration of resistin and UA, and SI value compared among SNV (−420G/C) genotypes and genotype groups in the studied population.

Parameter	Genotype	Total	Hypertensive (HT)	Non-Hypertensive (NHT)	High Salty Taste Preference (HS)	Normal Salty Taste Preference (NS)
Resistin (ng/mL)	CC	6.3 ± 2.5 113	6.04 ± 1.61 48	6.48 ± 3.0 65	6.28 ± 2.68 62	6.38 ± 2.33 49
	CG	7.06 ± 4 113	7.29 ± 4.55 60	6.8 ± 3.29 53	6.59 ± 2.67 49	7.9 ± 4.59 57
	GG	6.20 ± 2.88 31	6.63 ± 3.38 16	5.88 ± 1.31 15	6.42 ± 4.75 11	6.19 ± 1.25 19
Comparison	CC/GC/GG	*p* = 0.64 **	*p* = 0.82 **	*p* = 0.86 **	*p* = 0.25 **	*p* = 0.74 **
	GG + CG/CC	*p* = 0.50 *	*p* = 0.77 *	*p* = 0.54 *	*p* = 0.70 *	*p* = 0.86 *
Uric acid (mg/dL)	CC	5.80 ± 1.41 142	5.96 ± 1.51 62	5.68 ± 1.33 80	5.89 ± 1.45 74	5.75 ± 1.39 66
	CG	5.66 ± 1.29 138	5.96 ± 1.51 76	5.45 ± 1.43 62	5.69 ± 1.33 54	5.65 ± 1.22 75
	GG	5.65 ± 1.26 40	5.87 ± 1.29 21	5.40 ± 1.21 19	5.5 ± 1.32 17	5.74 ± 1.26 22
Comparison	CC/GC/GG	*p* = 0.76 **	*p* = 0.97 **	*p* = 0.49 **	*p* = 0.5 **	*p* = 0.9 **
	GG + CG/CC	*p* = 0.28 *	*p* = 0.49 *	*p* = 0.12 *	*p* = 0.16 *	*p* = 0.85 *
SI	CC	8.29 ± 2.57 115	8.80 ± 2.96 55	7.82 ± 2.07 60	8.11 ± 2.41 59	8.50 ± 2.76 55
	CG	8.52 ± 5.63 114	8.75 ± 4.39 67	8.19 ± 7.08 47	9.56 ± 8.21 48	7.57 ± 2.11 61
	GG	11.31 ± 17.22 34	9.12 ± 5.39 20	14.43 ± 26.41 14	15.51 ± 26.68 14	8.36 ± 2.38 20
Comparison	CC/CG/GG	*p* = 0.42 ^##^	*p* = 0.70 ^##^	*p* = 0.30 ^##^	*p* = 0.62 ^##^	*p* = 0.09 ^##^
	GG + CG/CC	*p* = 0.47 ^#^	*p* = 0.67 ^#^	*p* = 0.27 ^#^	*p* = 0.91 ^#^	*p* = 0.14 ^#^

SI: stiffness index; *N*: (number of patients); * Mann–Whitney U test; ^#^ T-student test; ^##^ analysis of variance (ANOVA), ** Kruskal–Wallis ANOVA; All other values are expressed as mean ± SD or percentage.

**Table 9:** The comparison of the serum concentration of resistin and UA, and SI within particular groups carrying GG + CG or CC genotypes.

Parameter	Groups	Hypertensive (HT)	Non-Hypertensive (NHT)	*p*	High Salty Taste Preference (HS)	Normal Salty Taste Preference (NS)	*p*
Resistin (ng/mL)	GG + CG	7.10 ± 4.39	6.68 ± 3.05	0.52 *	6.53 ± 3.01	6.87 ± 4.03	0.60 *
	CC	6.19 ± 2.06	6.44 ± 2.94	0.69 *	6.37 ± 2.81	6.38 ± 2.33	0.97 *
Uric acid (mg/dL)	GG + CG	5.83 ± 1.19	5.4 ± 1.4	0.03 *	5.58 ± 1.37	5.56 ± 1.23	0.69 *
	CC	5.97 ± 1.48	5.69 ± 1.31	0.36 *	5.92 ± 1.40	5.75 ± 1.38	0.46 *
SI	GG + CG	8.60 ± 3.41	9.98 ± 14.57	0.44 ^#^	10.85 ± 14.75	7.81 ± 2.20	0.04 ^#^
	CC	9.12 ± 4.80	7.75 ± 2.04	0.03 ^#^	8.41 ± 4.40	8.45 ± 2.75	0.95 ^#^

SI: stiffness index; * Mann–Whitney U test; ^#^ T-student test. All other values are expressed as mean ± SD or percentage.

**Table 10:** Significant correlations between resistin, UA, SI and other parameters evaluated in the study.

Parameter	Groups	r Coefficient *	Hypertensive (HT)	Non-Hypertensive (NHT)	High Salty Taste Preference (HS)	Normal Salty Taste Preference (NHS)
Resistin (ng/mL)	Both	SI (0.17)	-	SI (0.21)	*-*	*-*
	GG + CG	DBP (0.17)	-	DBP (0.29)	-	-
	CC	-	Age (0.29)	SI (0.29)	-	-
Uric acid (mg/dL)	Both	Age (0.17) BM (0.35) WC (0.36) SI (0.20) SBP (0.17) DBP (0.11)	BM (0.20) WC (0.23) SI (0.19)	Age (0.21) BM (0.45) WC (0.45) SBP (0.18)	Age (0.16) BM (0.43) WC (0.44) SI (0.26) SBP (0.23) DBP (0.17)	Age (0.16) BM (0.24) WC (0.28)
	GG + CG	Age (0.22) BM (0.29) SI (0.24) SBP (0.23)	SI (0.24)	Age (0.27) BM (0.40) SBP (0.28)	BM (0.29) SI (0.40)	BM (0.23) SBP (0.24)
	CC	BM (0.40) DBP (0.18)	BM (0.30) DBP (0.28) WC (0.33)	BM (0.50) WC (0.54)	BM (0.54) DBP (0.28)	BM (0.24)
SI	Both	Age (0.35) WC (0.15) Res (0.17) SBP (0.24) DBP (0.17) UA (0.20)	Age (0.31) UA (0.19)	Age (0.37) Res (0.21) SBP (0.24)	Age (0.46) WC (0.29) SBP (0.35) DBP (0.19)	Age (0.26)
	GG + CG	Age (0.40) SBP (0.30) UA (0.24)	Age (0.27) UA (0.24)	Age (0.54) SBP (0.35)	Age (0.65) SBP (0.40) UA (0.40)	SBP (0.23) DBP (0.24)
	CC	Age (0.28)	Age (0.35)	Res (0.29) Age (0.25)	Age (0.29) SBP (0.32) DBP (0.21)	Age (0.26)

SI: stiffness index; UA: uric acid; BM: body mass; WC: waist circumference; SBP: systolic blood pressure; DBP: diastolic blood pressure; Res: resistin; * Spearman Rank correlation, r: correlation coefficient (rho).

## Data Availability

Not applicable.

## Supplementary Materials

The following supporting information can be downloaded at: https://www.mdpi.com/article/10.3390/nu14091789/s1, Table S1: Genotype frequencies of SNV (−420G/C) in women and men group.
