# Supplementary material for: The Association between Serum Resistin Level, Resistin (−420C/G) Single Nucleotide Variant, and Markers of Endothelial Dysfunction, including Salt Taste Preference in Hypertensive Patients
Source: Nutrients. 2022 Apr 24;14(9):1789. doi: 10.3390/nu14091789 (PMC9102533; doi:10.3390/nu14091789)
Supplement: Supplementary file 1 [file nutrients-14-01789-s001.zip › nutrients-1684593-supplementary.pdf]

Table S1. Genotype frequencies of SNV (-420G/C) in women and men group.

	Women			Men		
**Genotype**	**Hypertensive (HT)**	**Non-hypertensive (NHT)**		**Hypertensive (HT)**	**Non-hypertensive (NHT)**	
CC	40 (38.10%)	56 (49.12%)		22 (39.29%)	24 (49.98%)	
CG	54 (51.43%)	45 (39.47%)		24 (42.86%)	19 (38.78%)	
GG	11 (10.48%)	13 (11.40%)		10 (17.86%)	6 (12.24%)	
Comparison	χ^2	p-value	OR (95% CI)	χ^2	p-value	OR (95% CI)
CC/CG/GG	3.28	0.19	-	1.20	0.54	-
GG+CG/CC	1.75	0.18	1.43 (0.84-1.45)	2.62	0.1	1.89 (0.87-4.12)

p – statistical significance; OR – odds ratio; χ^2 test
